# Small Molecule Inhibitor of Type Three Secretion Suppresses Acute and Chronic *Chlamydia trachomatis* Infection in a Novel Urogenital Chlamydia Model

**DOI:** 10.1155/2015/484853

**Published:** 2015-01-28

**Authors:** Ekaterina A. Koroleva, Natalia V. Kobets, Egor S. Zayakin, Sergei I. Luyksaar, Ludmila A. Shabalina, Naylia A. Zigangirova

**Affiliations:** Gamaleya Institute of Epidemiology and Microbiology, Ministry of Health Russian Federation, Gamaleya Street 18, Moscow 123098, Russia

## Abstract

Previously, we reported that a compound from a group of thiohydrazides of oxamic acids, CL-55, possessed antichlamydial activity *in vitro* that was accompanied by a decreased translocation of the type three secretion effector, IncA, into the host cell. In this study, the antichlamydial activity of CL-55 was tested *in vivo* in DBA/2 mice infected with *C. trachomatis* serovar D. We found that intravaginal inoculation of DBA/2 mice with the clinically relevant strain, *C. trachomatis* serovar D, results in a course of infection and pathology similar to that observed in humans. The early stage of infection in this model was characterized by a shedding of *Chlamydia* in vaginal secretions followed by an ascending infection and inflammation in the upper genital tract. We found that CL-55 possessed antibacterial activity *in vivo* and was able to control *C. trachomatis* vaginal shedding, ascending infection, and inflammation in the upper genital organs in DBA/2 mice. Our data provide a proof of concept for the protective effect of the thiadiazinon, CL-55, against chlamydial infection *in vivo* and support the feasibility of further studies of its potential therapeutic applications.

## 1. Introduction

*Chlamydia trachomatis* is a leading cause of sexually transmitted diseases, with an estimated 100 million new cases reported annually [1]. This organism is responsible for a variety of genital syndromes in men and women, with infertility as an outcome in many cases [2].

Lack of an efficient antibacterial therapy for chronic chlamydia infection, as well as the rise of antibiotic resistance, underscores the importance of searching for new strategies of antibacterial treatment [3, 4]. Among the new approaches is the development of small molecule virulence inhibitors that can disarm bacteria and allow the host to control the infection.

Recently, we performed an identification of novel, small-molecule type three secretion (T3S) inhibitors by chemical modification of thiohydrazides of oxamic acids and experimental screening of their derivatives. Selected compounds from a group of thiadiazinons were assessed for antichlamydial activity* in vitro*. We demonstrated that these compounds inhibit chlamydial growth* in vitro* and that this was accompanied by the decreased translocation of the type three secretion effector, IncA [5]. These results prompted us to proceed to evaluation of new inhibitors in an animal model.

Human serovars of* C. trachomatis* have limited pathogenicity in mice. Thus, intravaginal infection in C3H/HeJ mice is used in chemotherapy studies [6] and allows for the study of bacterial shedding but not the upper genital tract pathology. Consistent establishment of infection in the upper genital tract of mice with human serovars of* C. trachomatis* requires hormonal manipulation and surgical intervention to place the organisms directly into the upper genital tract via the uterus or ovarian bursa. This procedure is time-consuming but results in salpingitis, altered tubal function, and impaired fertility [7, 8], and this model has been used successfully to evaluate new antimicrobial agents [9]. Infection can also be induced by intravaginal inoculation, but development of salpingitis from this route has been reported to be serovar dependent and irregular; this model would therefore be unsuitable for studying the long-term effects of chemotherapy on upper genital tract inflammation [10].

Most studies tend to use* C. muridarum* for chemotherapy and vaccine studies, which is genetically and antigenically distinct from human biovars [11, 12]. In addition, this strain was reported to respond differently to some antibacterial agents [13].

We report here, for the first time, the development of an upper genital tract infection in progesterone-treated, susceptible DBA/2 mice caused by intravaginal inoculation with clinically significant* C. trachomatis* serovar D, in which salpingitis and hydrosalpinx formation were observed. We also have expanded our work on the antibacterial activity of T3S inhibitors on chlamydial shedding, ascending upper genital tract chlamydial infection and inflammatory sequelae in susceptible DBA/2 mice and found that one T3S inhibitor efficiently suppressed both acute and chronic infection and ameliorated the upper genital pathology in an animal model.

## 2. Materials and Methods

### 2.1. Organisms

*C. trachomatis* serovar D D/UW-3/CX (ATCC VR 885) was grown in cycloheximide-treated McCoy cells using conventional methods. The growth medium was minimal essential medium supplemented with 10% fetal calf serum, 1% nonessential amino acids, 2 mM L-glutamine, 1% vitamins, and 0.5% glucose. Cell lysates containing approximately 3.5 × 10^4^ inclusion-forming units (IFU) per mL were prepared and stored at −70°C.

### 2.2. Animals

Female DBA/2 mice obtained from Animal Resource Center (Andreevka, Moscow region), weighing 14 to 16 g, were used throughout and were given access to food and water ad libitum.

### 2.3. Inoculation

The animals subcutaneously received 33 mg per kg of the progesterone analog, proligestone (Covinan, Intervet Schering-Plough Animal Health, Netherlands). Five days later, the mice were infected with 10^6^ CFU/mouse of* Chlamydia trachomatis* serovar D in 40 *μ*L of PBS. The control animals received sterile PBS.

### 2.4. Drug Treatment

Azithromycin was given as a daily, single, oral dose of 10 mg/kg. Treatment was administered orally for 5 days to mimic the regimen often recommended for treatment in humans.

4-(3-etoxy-4-hydrobenzil)-5-oxo-5,6-dehydro-4H-[1,3, 4]-thiadiazol-2-(2,4-biftorphenyl)-carboxamid, designated as CL-55, was given as an intraperitoneal injection of 50 or 100 mg/kg. Inhibitor solutions were prepared by mixing the active substance with Tween 80 and dissolving it in 1% starch in PBS. The control infected mice received a solution of 1% starch in PBS with Tween 80. The early effects of therapy were assessed by measuring vaginal shedding of chlamydiae starting at day three postinfection. The long-term therapy outcome was assessed using Real Time-PCR to measure chlamydial DNA in the ovaries and uteri 4 weeks after infection. Genital tissues from the infected mice, both treated and untreated, were processed for histopathological examination at days 28 to 30 after infection.

### 2.5. Isolation of Chlamydia

The canal and exocervix were vigorously scraped, and swabbed material was transferred into 2-sucrose phosphate transport medium and frozen immediately at −70°C. All specimens were processed on the same day. Thawed, undiluted samples were plated in 0.5 mL volumes onto McCoy cells on coverslips (12 mm diameter) in 24-well plates, and the infection was enhanced by centrifugation. Coverslips were incubated with FITC-conjugated monoclonal antibodies against chlamydial lipopolysaccharide (Nearmedic Plus, Russia) for 48 h. Inclusion-containing cells were examined and photographed with a Nikon Eclipse 50i fluorescent microscope at 1350x magnification. The results were expressed as lg IFU per milliliter of sample.

### 2.6. Real Time PCR

Samples for chlamydial DNA detection were collected in phosphate-buffered saline (PBS) and frozen at −20°C. DNA was extracted from excised genital tissues with an automated nucleic acid extractor, NucliSENS easyMAG (bioMérieux, Netherland). Chlamydial DNA was amplified with a* C. trachomatis* PB screen kit (Syntol, Moscow, Russia) and Real Time PCR cycler, CFX96 (Bio-Rad, USA).

### 2.7. Histopathology

Uteri and ovaries were frozen in the regimen of −60°C to −20°C temperature gradient in the electronic Cryotome Shandon (USA), and serial 8 *μ*m-thick sections were made. Sections were stained with haematoxylin and eosin (H&E) and examined without knowledge of the experimental group to which they belonged.

### 2.8. Statistical Analysis

All data were presented as the mean ± standard deviation (SD). All statistics were performed using GraphPad Prism version 6.00 (GraphPad Software Inc, California, USA). Significance was set at *P* < 0.05 for all tests.

## 3. Results

### 3.1. DBA/2 Mice Intravaginally Infected with* C. trachomatis* Serovar D Have Both Acute Chlamydia Shedding and Upper Tract Infection and Pathology

As existing* C. trachomatis* infection models in mice do not correspond to a human course of pathological sequelae, we developed a new murine* C. trachomatis* infection model. For this, we utilized the DBA/2 strain of mice that have a range of genetic defects that make them more susceptible to some bacterial infections and reproductive pathology [14, 15].

**Figure 1:**
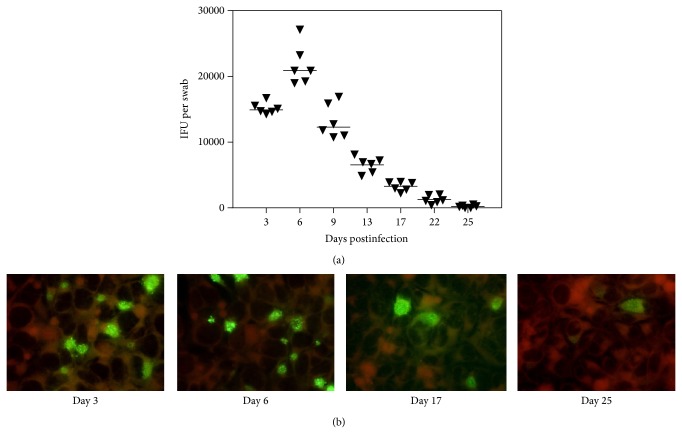
(a) Chlamydia inclusion-forming units (IFUs) obtained from vaginal swabs of DBA/2 mice infected with* C. trachomatis* serovar D. Shown are the IFU counts for vaginal swabs obtained at days 3 to 25. The median number of IFUs is indicated by the horizontal line. (b) Microphotographs of McCoy cell cultures infected with* C. trachomatis* from vaginal swabs of infected mice obtained at days 3 to 25.

In these studies, progesterone-treated DBA/2 mice received 10^6^ IFU of* C. trachomatis* serovar D intravaginally. Three days later, a mean count of ~10^4^ IFU/mL was recovered from vaginal swabs from DBA/2 mice (Figure 1). We registered shedding until day 25 when it was 10^2^ IFU/mL, though infection partly ascended at that time and could also be registered in the upper genital organs.

**Figure 2:**
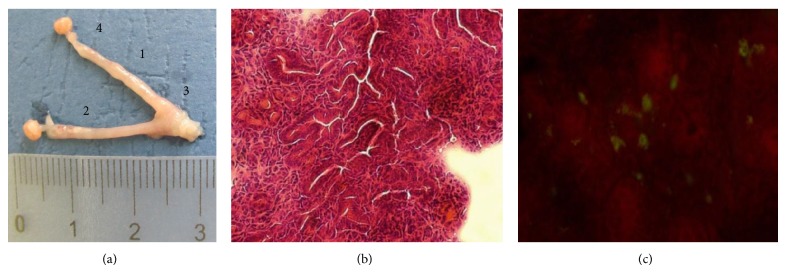
Features of pathology of the upper genital organs after intravaginal infection of DBA/2 mice with* C. trachomatis* serovar D. (a) Uterine horns of infected mice at day 28 postinfection (1: hydrosalpinx, 2: endometritis, 3: endocervicitis, 4: oophoritis). (b) Uterine tissue of infected mice. (c)* C. trachomatis* inclusions in uterine tissue of infected mice.

Examination of the upper genital organs at day 28 postinfection revealed inflammation of uterine horns, mild hydrosalpinx, some features of endometritis, endocervicitis, and oophoritis (Figure 2(a)). Development of hydrosalpinx occurred in more than 80% of the mice after 28 days postinfection.

Histological examination of oviducts 28 days after infection revealed swelling of the subepithelial connective tissue, oviduct stenosis, and vascular impairments.

Numerous polymorphonuclear leukocytes were also evident (Figure 2(b)). Additionally,* Chlamydia* inclusions were found in the uterine tissue of the infected mice (Figure 2(c)).

**Figure 3:**
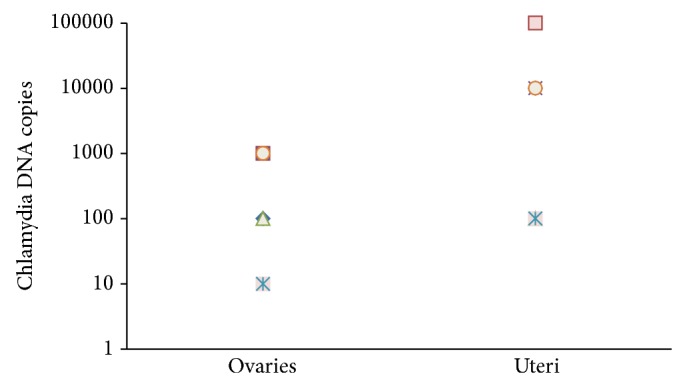
Chlamydial DNA detection in ovaries and uteri of* chlamydia*-infected mice at day 28 postinfection. Individual values for each mouse per group are shown.

The presence of bacteria in the upper genital tract was assessed using PCR. Chlamydial DNA was detected in the ovaries and uteri at day 28 postinfection, and thus, the PCR results confirmed the development of chronic urogenital infection in DBA/2 mice infected with clinically important* C. trachomatis* serovar D strain (Figure 3).

### 3.2. T3S Inhibitor Affects the Course of Infection* In Vivo*

As CL-55 showed promising results in the suppression of* C. trachomatis* infection* in vitro* [5], in this study we analyzed its antibacterial activity* in vivo* in DBA/2 mice. To this end, we infected progesterone-treated DBA/2 mice with 10^6^ IFU of* C. trachomatis* serovar D. To assess the inhibitor activity against acute infection, we treated mice with 50 mg/kg of CL-55 for 5 days starting from day 1 of infection (early therapy). To assess the inhibitor activity against chronic infection, we treated the mice with 50 or 100 mg/kg of CL-55 during 5 days starting from day 15 postinfection (late therapy). The efficiency of our inhibitor was compared to the efficiency of azithromycin used either as an early (day 1 postinfection) or late (day 15 postinfection) treatment.

**Figure 4:**
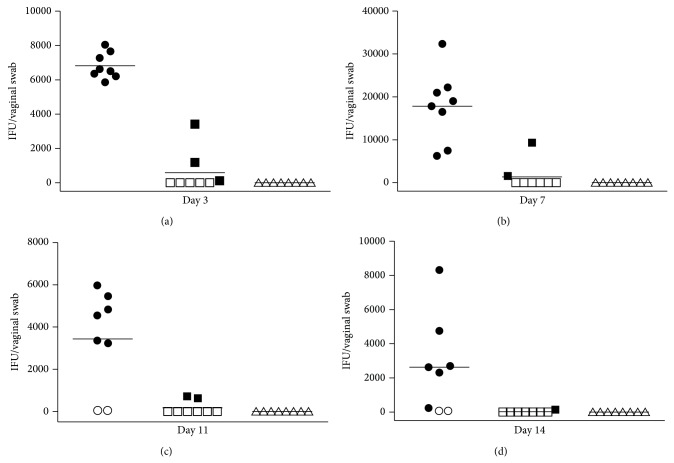
Chlamydia inclusion-forming units (IFUs) obtained from vaginal cultures for control (● or ◯, *n* = 8), CL-55-treated (■ or □, *n* = 8), and azithromycin-treated (▲ or △, *n* = 8) mice. Shown are the IFU counts for vaginal cultures obtained at the 3rd, 7th, 11th, and 14th days. The median number of IFUs is indicated by the horizontal line. Culture-negative (◯, □, and △) mice and culture-positive (●, ■, and ▲) mice are shown in each group.

To determine the short-term efficacy of each therapy, vaginal swabs were taken from the DBA/2 mice infected with 10^6^ IFU of* C. trachomatis* serovar D and treated with 50 mkg/kg of CL-55 starting from day 1 postinfection. Vaginal swabs were taken from days 3 to 14 following infection. The mice infected with* Chlamydia* without treatment or the mice treated with azithromycin were used as controls. All animals treated with azithromycin were culture negative (Figure 4). We also found a significant reduction of chlamydia inclusions at day 7 after infection in the mice treated with CL-55 starting from day 1. The level of inclusions dropped to zero in this group at day 11 postinfection (Figure 4). Thus, the effectiveness of our inhibitor in the treatment of acute infection was confirmed in these experiments.

**Figure 5:**
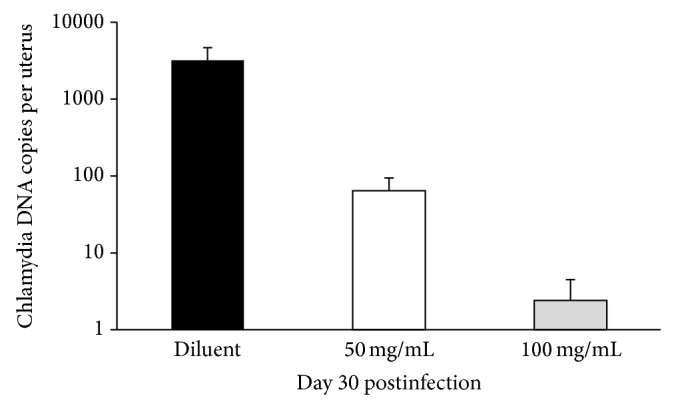
CL-55 treatment decreases the levels of* C. trachomatis* DNA in upper genital organs of DBA/2 mice infected with* C. trachomatis* serovar D. Black bar, infected DBA/2 mice treated with *растворит*e*ль*; light bar, mice treated with 50 mg/mL of CL-55; grey bar, mice treated with 100 mg/mL of CL-55. ^*^ *P* ≤ 0.05.

To assess the long-term effects of the therapy, we analyzed the ability of our inhibitor to suppress infection in the upper genital organs. To this end, we analyzed the levels of chlamydial DNA in uteri of the infected mice at day 30 postinfection. In this set of experiments, the mice were infected with 10^6^ IFU of* C. trachomatis* serovar D and treated with either 50 or 100 mg/kg of CL-55 intraperitoneally for 5 days starting from day 15 postinfection. Treatment of the infected mice with T3S inhibitor CL-55 resulted in a decrease of* C. trachomatis* DNA in the upper genital tract of these mice, and this effect was more pronounced in the group treated with 100 mg/kg of inhibitor (Figure 5). Thus, we confirmed that the T3S inhibitor CL-55 successfully inhibited chronic* C. trachomatis* infection* in vivo*.

As we have previously reported that CL-55 has minimal toxicity when tested* in vitro* using McCoy cells [5], in this study, we assessed the effects of this compound on the upper genital tract pathology. For this, the progesterone-treated mice were infected with 10^6^ IFU of* C. trachomatis* serovar D and were inoculated i.p. with 100 mkg/kg of CL-55 or diluent either at day 1 or at day 15 following infection. The mice were given a daily treatment of T3S inhibitor for 5 days, and 2 pairs of infected control and CL-55 treated mice were sacrificed at day 30 postinfection.

**Figure 6:**
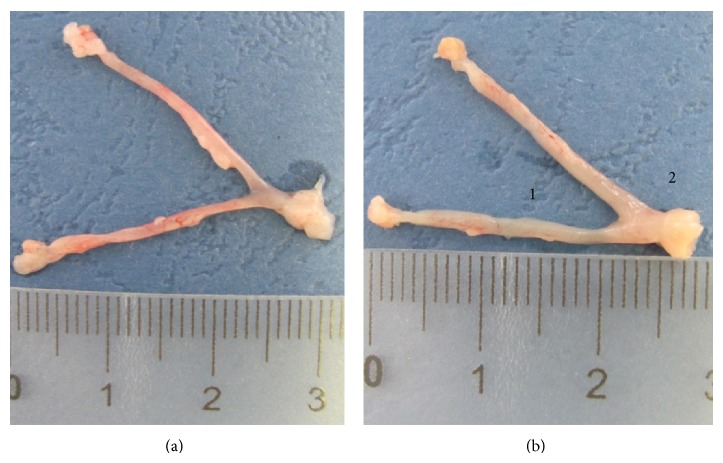
Treatment with CL-55 reduces uterine pathology in DBA/2 mice infected with* C. trachomatis* serovar D. Uterine horns of infected mice at day 30 postinfection are shown. (a) Mice treated with CL-55 from day 1 postinfection. (b) Mice treated with CL-55 from day 14 postinfection (1: endometritis, 2: endocervicitis).

We did not observe signs of pathology in the mice treated with the T3S inhibitor starting from day 1 (Figure 6(a)). Mild salpingitis and inflammation of endocervix were still observed in the group treated with CL-55 starting from day 15; however, we did not observe hydrosalpinx in this group (Figure 6(b)). Thus, both early and late treatment with CL-55 decreased the features of pathology in upper genital organs of the DBA/2 mice infected intravaginally with* C. trachomatis* serovar D. Mild upper genital tract pathology was also observed in groups treated with azithromycin (data not shown).

## 4. Discussion

Although* C. muridarum* reproduces human infection and its inflammatory sequelae in the upper genital tract of mice, it does not always share the* C. trachomatis* pattern of response to some antibacterial agents [13].

In this study, we show that intravaginal inoculation of DBA/2 mice with the clinically relevant* C. trachomatis* serovar D gives a course of infection and pathology similar to that observed in humans. The early stage of infection in this model was characterized by the shedding of* Chlamydia* in vaginal secretions, with numbers gradually declining over a 4-week period. After this time, no organisms could be detected. The later stage began from 4 to 5 weeks after infection when chlamydial DNA was detected in uteri and ovaries. This was accompanied by uterine horn inflammation, endometritis, endocervicitis, hydrosalpinx, and oophoritis detected in a majority of the mice. The course of events in this model was similar to that observed after intravaginal inoculation with* C. muridarum* or inoculation of human serovars by the technically more demanding intrauterine or intrabursal routes [7, 8]. Thus, many of the sequelae of chlamydial infection in women have been reproduced relatively simply in our mouse model. Moreover, these findings represent a significant contrast to other reports on the effect of intravaginal inoculation with* C. trachomatis*, in which the infection was described as mild and restricted only to the lower genital tract [6, 16].

As we have previously shown,* C. trachomatis* was susceptibly to our novel T3S inhibitor* in vitro* [5], and in this report, we have further extended our study to evaluate the susceptibility of* C. trachomatis* to our T3S inhibitor* in vivo*. To assess the efficacy of treatment with the T3S inhibitor in both the early and later stages of infection, we evaluated* Chlamydia* vaginal shedding,* Chlamydia* DNA in ovaries and uteri, and upper genital tract pathology after early (from day 1) or late (from day 15) treatment with our inhibitor. Chlamydial shedding was abrogated in the group receiving early treatment by day 14 (Figure 4). This was also accompanied by abrogation of the upper genital tract pathology (Figure 6(a)) in this group of mice. A decrease in the number of Chlamydial DNA copies was observed in the late treated group, but though the upper genital tract pathology was reduced in this group, it was still apparent, which could be either due to the persistence of low numbers of organisms or because the onset of inflammation was triggered before the treatment. Previously, Slepenkin and colleagues reported on the antibacterial activity of another T3S inhibitor, salicylidene acylhydrazide compound, in intravaginal chlamydial infection. In this study, the T3S inhibitor was tested as a microbicide and decreased shedding of* Chlamydia* in C3H/HeJ mice intravaginally infected with* C. trachomatis* serovar D [6]. Later, the same group assessed the antibacterial activity of two salicylidene acylhydrazide compounds administered systemically and again confirmed the efficacy of the chosen T3S inhibitors in the control of* Chlamydia* vaginal shedding [17]; however, the impact of the compound on the infection of the upper genital tract was not studied. In our study, we confirmed for the first time that the antibacterial activity of a T3S inhibitor can be extended to the control of ascending* C. trachomatis* infection and accompanying inflammatory sequelae in the upper genital organs.

## 5. Conclusions

Our data provide a proof of concept for the protective effect of one of the thiadiazinons, CL-55, against chlamydial infection* in vivo* and support the feasibility of further studies to test its potential in therapeutic applications.
